# The Influence of Human Connections and Collaboration on Research Grant Success at Various Career Stages: Regression Analysis

**DOI:** 10.2196/49905

**Published:** 2024-02-28

**Authors:** Akiko Hashiguchi, Makoto Asashima, Satoru Takahashi

**Affiliations:** 1 Institute of Medicine University of Tsukuba Tsukuba Japan; 2 Advanced Comprehesive Research Organization (ACRO) Teikyo University Itabashi Japan

**Keywords:** biomedical researchers, grant success, human connection, peer researchers, synergistic collaborations, research development

## Abstract

**Background:**

Documenting the grant acquisition characteristics of a highly selective group of researchers could provide insights into the research and faculty development of talented individuals, and the insights gained to foster such researchers will help university management strengthen their research capacity.

**Objective:**

This study examines the role of human connections in the success of biomedical researchers in Japanese universities.

**Methods:**

This study used grant data from the Grants-in-Aid for Scientific Research (GIA) program, the largest competitive research funding program in Japan, to collect information on projects and their implementation systems obtained throughout the participants’ careers. Grant success was measured by the number and amounts of the awards obtained while participants occupied the role of principal investigator. Human connections were quantified by the number of projects in which the participants took part as members and were classified by their relationship with the project leader. Data were matched with information on career history, publication performance, and experience of the participants with government-funded programs apart from GIA and were analyzed using univariate and multivariate regression analyses.

**Results:**

Early-career interpersonal relationships, as measured using the *h*-index value of the researchers who provided the participants with their initial experience as project members, had a positive effect on grant success. The experience of contributing to prestigious research programs led by top researchers dramatically increased the cumulative amount of GIA awards received by the participants over time. Univariate logistic regression analyses revealed that more interactions with upper-level researchers resulted in fewer acquisitions of large programs (odds ratio [OR] 0.67, 95% CI 0.50-0.89). Collaboration with peers increased the success rate of ≥2 research grants in large programs in situations in which both the participant and project leader were professors (OR 1.16, 95% CI 1.06-1.26). Tracking the process of research development, we found that collaboration during the periods of 10 to 14 years and 15 to 19 years after completing a doctorate degree determined the size of the project that the participant would obtain—interactions with peer researchers and subordinates during the 10- to 14-year postdegree period had positive effects on ≥2 large-program acquisitions (OR 1.51, 95% CI 1.09-2.09 and OR 1.31, 95% CI 1.10-1.57, respectively), whereas interactions with subordinates during the 15- to 19-year postdegree period also had positive effects (OR 1.25, 95% CI 1.06-1.47). Furthermore, relationships that remained narrowly focused resulted in limited grant success for small programs.

**Conclusions:**

Human networking is important for improving an individual’s ability to obtain external funding. The results emphasize the importance of having a high-*h*-indexed collaborator to obtain quality information early in one’s career; working with diverse, nonsupervisory personnel at the midcareer stage; and engaging in synergistic collaborations upon establishing a research area in which one can take more initiatives.

## Introduction

### Background

The university sector has played a major role in the production of scientific knowledge by facilitating research based on the unique conception of researchers and has supported important societal benefits by helping companies source innovation [1]. As the world becomes increasingly unstable, the university sector will be required to create more knowledge and supply more human resources, leading to breakthrough innovation. However, as knowledge accumulates and science becomes more specialized, fewer remarkable discoveries are made [2]. In addition, conducting research that answers simple but major questions is becoming difficult under the recent system of university performance evaluation, which emphasizes short-term outcomes against a backdrop of the demand for accountability in the allocation of public funds [3,4]. This emphasis on short-term results is significant from the perspective of university management in countries where public funds are the primary source of university revenue. For example, Japanese universities need to equip faculty members with strong research skills to receive more government resource allocation, especially after 2019, when performance-based allocations were introduced [5].

High competitiveness in research is measured by the number and impact of publications and the acquisition of external funding; the latter is particularly important from the perspective of university management. Importantly, in the long run, the ability to draw a vision will influence the future direction of the research field and attract young researchers. This innovative power—that is, creating a common understanding that a research field that did not previously exist will exist in the future—is expected to materialize as large research projects supported by significant funding. Therefore, it is important to understand how personnel are capable of obtaining vast amounts of external funding. By examining the existing funding framework in Japan, this study observed that the larger the funding program, the more broadly the disciplines are grouped to form review committees. Therefore, project leaders must convince reviewers from diverse fields of the significance of a project [6]. Project leaders of a group of researchers spanning multiple disciplines are required to be capable of discussing the worldview that the research can present.

Factors such as the exchange of ideas and expansion of knowledge are important for fostering visionary researchers. Accordingly, the effectiveness of international mobility and academic industry collaboration has been evaluated from the perspectives of higher education, research, and innovation policy [7-9]. In addition, the literature has emphasized the importance of “productive interactions” between researchers and social actors in studies that assess the social impact of scientific research [10,11]. These examples demonstrate that learning from others regarding perspectives beyond the scope of one’s research can lead to innovative ideas. However, few studies have focused on the impact of such “productive exchanges” on the human resource development of individual researchers.

Chan et al [12] analyzed the coauthorship patterns of Nobel laureates and found that encounters of heterogeneous ideas from different researchers, which occur early in the collaboration life cycle, generate the most innovative ideas that emerge from the collaborative relationship. This cross-fertilization of ideas is considered effective not only in the research development process of Nobel laureates but also in the process of cultivating creativity among researchers in general. Studies on creativity have shown that acting as a broker of valuable knowledge (ie, having a high level of “betweenness centrality” in a network) can facilitate information flow so as to generate new ideas [13]. A study of young researchers in biomedical sciences showed that effective collaboration with “nonsupervisor” peers is important for learning [14]. In the academic community, where the culture of apprenticeship is strong, handing over a research theme between a supervisor and an apprentice and taking over a network of people working on such a theme are effective methods for increasing one’s visibility in the field. In this context, how researchers relate not only to their immediate supervisors but also to their colleagues and researchers in other institutions is key to improving their competitiveness in grant acquisition, as previously demonstrated using selective groups of researchers with high betweenness centrality [15].

### Objectives

This study explored the objective metrics of success in grant acquisition using information from Grants-in-Aid for Scientific Research (GIA) projects and focusing on interpersonal relationships. By doing so, we aimed to identify the requirements for developing researchers capable of winning external funding for university management and who, ultimately, can influence research trends through innovative ideas and high-level capabilities in project realization.

## Methods

### Overview

In Japan, GIA provides fundamental financial assistance for academic research activities that cover all fields with the objective of promoting scholarship and advancing creative research [6]. It is the largest competitive research funding program in Japan, which began in the 1950s. Even after the introduction of government funding programs for various purposes, it continues to account for most funds that support “bottom-up” researcher-led projects [16,17]. GIA includes basic programs (classified according to the amount awarded), innovation programs that take on new challenges, and specially promoted research programs that support outstanding original research that pioneers new scholarship. Other programs are also available to young researchers. Screening of GIA projects through peer review, although the breadth of the reviewers’ fields varies according to the program size [6]. Therefore, GIA award acquisition performance is considered an indicator that quantifies the academic creativity of researchers who have reached a certain level regardless of external conditions such as top-down policy requirements. This study focused on the implementation structure and member composition of projects supported by GIA.

### Data Sources

The grant acquisition history of the participants, that is, the research projects in which they were involved, was obtained from the GIA database, wherein participants’ names can be used as search terms to obtain information on their positive GIA awards (National Institute of Informatics, Japan) [18]. Although the GIA system has undergone several modifications and programs have been established or abolished over time, we focused on programs that most researchers currently in professorial positions are familiar with. Information on rejected projects was not included in this study.

Data on degree types were obtained from the doctoral dissertation database [19]. These data were introduced as an alternate indicator for understanding the effect of age as the exact age of each researcher is not publicly available. This indicator discriminates whether a degree was obtained by either enrolling in a graduate school and submitting a dissertation or submitting a dissertation and passing an examination even if the person was not enrolled in a graduate school (doctorate obtained by thesis). The latter is a unique system in Japan, and the degrees of this type are primarily awarded to people who conducted their research as employees of a company.

The productivity, contributions, and research impact of each researcher, as measured by the citation counts, were calculated from the publication history data obtained from the SciVal database under the license of the University of Tsukuba using the Scopus IDs as the search key (Elsevier) [20]. Factors that could not be determined through the aforementioned indicators were examined using a database that covered most government funding programs by entering the participants’ names (BIOIMPACT, Japan) [21].

### Ethical Considerations

This study did not require ethics approval as it did not collect data from human or animal participants. The names of the professors, external funding obtained, and publication records are publicly available. The 2 databases of grants were constructed to ensure transparency in public research funding allocation and allow users to search for the latest research information in Japan—one records the GIA program of the Ministry of Education, Culture, Sports, Science, and Technology of Japan [22] and the other records other government funding programs of the Cabinet Office; Ministry of Internal Affairs and Communications; Ministry of Health, Labour, and Welfare; Ministry of Agriculture, Forestry, and Fisheries; Ministry of Economy, Trade, and Industry; Ministry of Land, Infrastructure, Transport, and Tourism; Ministry of the Environment; and Ministry of Defense [23]. The analysis plan was not preregistered as this was a secondary analysis of open data extracted from public databases.

### Researcher Characteristics

This study considered the following characteristics in terms of the researchers’ competitiveness: the sum of the maximum allocated amount of projects obtained as principal investigators, total allocation earned as a principal investigator during the first 10 years after obtaining a PhD, and the number of projects obtained as principal investigators in large programs, such as Grant-in-Aid for Specially Promoted Research and Grant-in-Aid for Scientific Research (S), or small programs, such as Grant-in-Aid for Scientific Research (C). In terms of their personal attributes, we considered the number of years since obtaining a doctoral degree, whether the doctorate was obtained by thesis, sex, experience in nonuniversity institutions, the rankings of the university in which the researcher obtained their doctorate, and where the researcher is currently affiliated. To measure each researcher’s productivity, we considered the number of papers published, first-authored papers, and last-authored papers.

To clarify the effect of human connections, we included the following attributes: the number of coresearchers connected with through GIA projects throughout their career, the betweenness centrality score based on people-to-people connections in GIA projects, the *h*-index of the researcher with whom the participant researcher first became a member of GIA projects, the number of government-funded programs participated in as a project member, and the total *h*-indexes of the researchers with whom the participant researcher interacted through other government-funded programs. To measure the researchers’ interpersonal relationships, we considered the number of researchers who designated the participants as their GIA project members (upper level, peer, or subordinate) throughout their careers. We also considered the number of projects a researcher participated in during each period after obtaining a doctorate (10-14 y and 15-19 y after the doctorate), categorized by the relationship of the project leader to the participant (upper level, peer, or subordinate).

### Samples and Analysis Method

#### Analysis 1

This study focused on a single area of biomedical science to obtain detailed microstructural data on the relationship between research implementation and subsequent improvements in research conception skills. Biochemistry was selected as the target field as it is the basis of today’s medicine given its focus on molecular mechanisms and as the 54 professors in this field include 5 (9%) awardees of Grant-in-Aid for Specially Promoted Research or Grant-in-Aid for Scientific Research (S) and 16 (30%) awardees of Grant-in-Aid for Scientific Research (A). These numbers exceed those in the field of hematology, with 3% (1/35) of awardees of Grant-in-Aid for Specially Promoted Research or Grant-in-Aid for Scientific Research (S) and 9% (3/35) of awardees for Grant-in-Aid for Scientific Research (A), and dermatology, with 5% (2/39) of awardees for Grant-in-Aid for Specially Promoted Research or Grant-in-Aid for Scientific Research (S) and 18% (7/39) of awardees for Grant-in-Aid for Scientific Research (A). This indicates that biochemistry is a suitable field for analyzing the competitiveness of researchers in Japan.

The names of the researchers who held professorships in biochemistry at the medical schools of Japanese universities between 2020 and 2022 were extracted from the websites of each university. In total, the study identified 54 researchers, including 2 (4%) female researchers, from 11 schools belonging to the top university group and other national universities. Academic and professional histories were obtained from their websites. In terms of education, 72% (39/54) graduated from schools of medicine, 87% (47/54) obtained PhDs in medicine, and 69% (37/54) obtained their bachelor’s and doctorate degrees in the same university. The mean number of years since receiving the degree was 29 (SD 6). We extracted 1473 GIA projects, of which 803 (54.51%) were implemented by the 54 participants as principal investigators (Table 1). For analysis 1, we summed the maximum allocated amount that each researcher won as the principal investigator and used it as the dependent variable. The independent variables were calculated using data obtained from each source. We controlled for sex (*female*) and experience at nonuniversity institutions (*nonuniversity institutions*). In this analysis, *nonuniversity institutions* refers to corporations and their associations. A lagged variable was designated for the total allocation of projects won by the participants as principal investigators for 10 years after each researcher obtained a PhD. Finally, researchers with missing data were excluded, and 52 were included in the analysis. Multiple regression analysis was performed using the XLSTAT statistical software (Addinsoft).

**Table 1:** Data on the researcher population used in the study and their characteristics.

	Analysis 1	Analysis 2
Participants, N_1_	52	982
Female participants, N_1,_ n (%)	2 (3.8)	55 (5.6)
Number of projects obtained by the participants^a^, N_2_	1473	11,437
Number of projects obtained by the participants as PI^a,b,c^, N_2_, n (%)	803 (54.51)	4381 (37.43)
Number of projects participated in as project member^a,b^, N_2_, n (%)	710 (48.20)	7056 (61.69)

^a^The count includes duplicates because of, for example, changes in principal investigator. N_1_ is the number related to participants and N_2_ is the number related to acquired projects.

^b^The analysis includes Grant-in-Aid for Scientific Research (S), (A), (B), or (C); Grant-in-Aid for Specially Promoted Research; and Grant-in-Aid for Young Scientist (S) and (A) for analysis 2.

^c^PI: principal investigator.

#### Analysis 2

We obtained people-to-people connections up to the third level by counting 54 participants based on the team member list of projects throughout their careers. The researchers extracted in the second tier of connections, that is, those who had primary relationships with the 54 participants, totaled 982 and conducted 11,437 projects, of which 4381 (38.31%) were implemented by the 982 participants as principal investigators (Table 1). The types of colleagues were analyzed using the data of the research members for each project. We categorized the researchers who designated the participants as their project members based on whether they were upper-level or peer researchers of the participants. If they were peer researchers, we further categorized the person according to position rank. Connections with the participant were then classified according to their relationship. Analysis 2 focused not only on the totaled highest amount allocated for each project r of projects but also on the type of programs won. The number of large or small projects won as principal investigators was used as the dependent variable. Furthermore, we categorized the projects in which each participant took part as a project member according to the period in which each of them obtained a PhD to obtain information on when interactions are important over time. Principal component analysis and multiple regression analysis were conducted using the XLSTAT statistical software.

## Results

### Analysis 1

**Figure 1:**
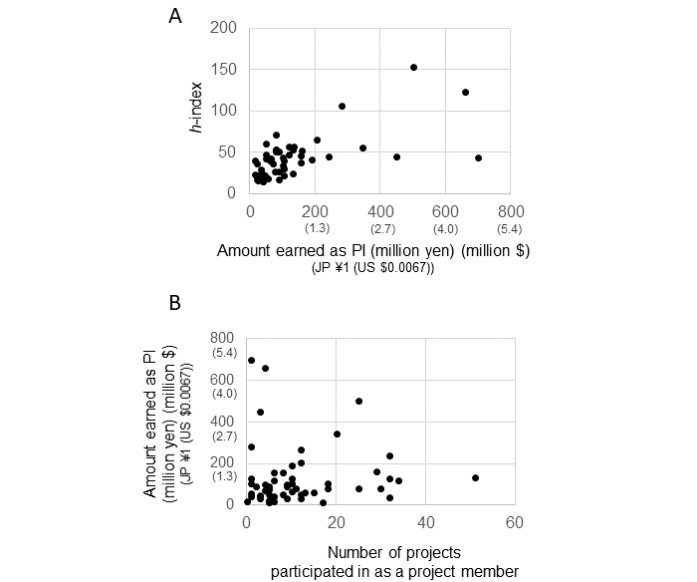
(A) Regression of the *h*-index value against the amount allocated to each participant as principal investigator (PI; includes Grants-in-Aid for Scientific Research, Grants-in-Aid for Specially Promoted Research, and Grants-in-Aid for Young Scientists). (B) Regression of the amount allocated to each participant as PI against the number of projects in which they participated as project members (N=52).

Multimedia Appendix 1 presents the descriptive statistics of the variables used in the analysis. When we regressed the totaled highest amount allocated for each project of the 52 selected researchers on their *h*-index, which represents the overall competitiveness of a given researcher with respect to publications, we found a positive correlation (Figure 1A). This result suggests that the variable defined previously is consistent with a researcher’s perceived competitiveness. The relationship between the dependent variable and the number of projects in which each researcher participated as a project member is shown in Figure 1B. We analyzed the determinants that contributed to success in obtaining GIA awards as principal investigators and focused on interpersonal relationships in the projects in which they participated as project members (models 1-4 in Table 2).

**Table 2:** Probability of grant success (based on a 2-tailed t test; ordinary least squares was used for analysis; N=52)^a^.

	Model 1	Model 2	Model 3	Model 4
Number of years since doctoral degree, unstandardized coefficient (SE)	0.08 (0.10)	0.14 (0.10)	0.08 (0.10)	0.09 (0.10)
Female (no), unstandardized coefficient (SE)	0.10 (0.09)	0.09 (0.08)	0.09 (0.09)	0.08 (0.08)
Female (yes), unstandardized coefficient (SE)	0 (0)	0 (0)	0 (0)	0 (0)
Nonuniversity institutions (no), unstandardized coefficient (SE)	−0.57^b^ (0.09)	−0.50^b^ (0.10)	−0.55^b^ (0.09)	−0.54^b^ (0.09)
Nonuniversity institutions (yes), unstandardized coefficient (SE)	0 (0)	0 (0)	0 (0)	0 (0)
University rank (graduate)^c^, unstandardized coefficient (SE)	—^d^	—	0.01 (0.09)	—
University rank (currently affiliated)^c^, unstandardized coefficient (SE)	—	—	−0.17 (0.11)	—
Total allocation in early stage^e^, unstandardized coefficient (SE)	—	0.20 (0.11)	—	—
Number of coresearchers connected with through projects, unstandardized coefficient (SE)	-0.47^f^ (0.19)	−0.49^f^ (0.18)	−0.48^f^ (0.19)	−0.32^f^ (0.14)
Betweenness centrality^g^, unstandardized coefficient (SE)	0.28 (0.24)	0.38 (0.24)	0.32 (0.24)	—
*h*-index of the researcher with whom the participant first became a project member, unstandardized coefficient (SE)	0.19^f^ (0.09)	0.18^f^ (0.09)	0.13 (0.10)	0.16 (0.08)
Number of papers, unstandardized coefficient (SE)	−0.16 (0.24)	−0.15 (0.24)	−0.22 (0.25)	—
Number of last-authored papers, unstandardized coefficient (SE)	0.67^h^ (0.24)	0.55^f^ (0.24)	0.65^f^ (0.24)	0.65^b^ (0.13)
*F* test (*df*)	12.68^b^ (8, 43)	12.30^b^ (9, 42)	10.57^b^ (12, 39)	16.88^b^ (6, 45)
Adjusted *R*^2^	0.647	0.666	0.652	0.651
AIC^i^	954.178	952.072	954.918	951.888

^a^The dependent variable was the sum of the highest amount allocated for each project number of projects obtained as principal investigator. This included Grant-in-Aid for Scientific Research (S), (A), (B), or (C); Grant-in-Aid for Specially Promoted Research; and Grant-in-Aid for Young Scientists (S) or (A).

^b^*P*<.001.

^c^These variables were quantified based on The Times Higher Education World University Rankings 2023 and have 7 levels of classification, with a higher ranking having a smaller numerical value, that is, 1 for the top 100 ranking, 2 for 201 to 600, a value of 3 for 601 to 1000, a value of 4 for 1001 to 1200, a value of 5 for 1201 to 1500, a value of 6 for ≤1501, and 7 for out of ranking.

^d^Variables were not included in the model.

^e^The total allocation earned as principal investigator during the first 10 years after obtaining a PhD.

^f^*P*<.05.

^g^The Cytoscape software (version 3.4.0; Cytoscape Team) was used to calculate the value using people-to-people connections up to the third level starting from the participant based on the team member lists of all GIA projects throughout their career.

^h^*P*<.01.

^i^AIC: Akaike information criterion.

Model 1 is the base model that includes the researchers’ attributes, human connections in the history of GIA grant success, and research performance. The effect of human connections was examined using networking level as measured using the value of betweenness centrality and quality of connections expressed through the *h*-index of the coresearcher. To examine the effects of publication performance, we included the number of papers and last-authored papers published. Model 2 controls for early career success in obtaining GIA awards to mitigate endogeneity, and model 3 includes variables for university rank at various stages in the researchers’ career. Model 4 is a simplified model with variables that were found to be highly influential throughout the analysis. Regarding the control variables, the coefficients of not having experience at nonuniversity institutions were significantly negative (*P*<.001). Difference based on sex exhibited no significant effect, but the number of female researchers in the analysis was small; thus, this result is not definitive. Considering research performance, the impact of last authorship was large (models 1-4).

Regarding human connections, researchers with fewer collaborative partners tended to win more grants; however, this trend was inconsistent with the correlation coefficients shown in Multimedia Appendix 1. Betweenness centrality, which indicates the visibility of each researcher within the collaborative network, did not exert a significant effect on lifetime grant acquisition (models 1-4). The *h*-index value of the researcher who first invited the participant to become a project member had a significant positive impact on the participant’s grant success (model 1). The effect of these 3 variables remained significant even after incorporating variables related to grant success in the early stages of the researchers’ career (model 2). The correlation between university rank and grant success shows that researchers affiliated with higher-ranked universities tend to be more successful (Multimedia Appendix 1); however, when considering the regression models, the rankings of the universities where a researcher obtained their PhD degree and where they were currently working did not, by themselves, affect grant success. When university ranks were incorporated into the regression model, the effect of the *h*-index value of the researcher who first invited the participant to become a project member was not significant (model 3). Overall, the result of the *h*-index value indicates the importance of having a good research guide as the first step but also suggests that a researcher’s grant success can be affected by the new connections established during career development. Finally, model 4, which retains this variable together with last authorship and number of collaborative partners, has the smallest Akaike information criterion value and was considered the best-fit model.

Initially, we assumed that the researchers who first invited the participants to become a project member were laboratory heads and direct superiors, but this was not always the case (Table 3). Only 29.6% (32/108) of the researchers were direct superiors, 5.5% (6/108) were subgroup heads other than professors, and 7.4% (8/108) were peers or subordinates in their own institution. Notably, 8.3% (9/108) were external upper-level professionals (ie, from other institutions; Table 3). The correlation matrix in Multimedia Appendix 1 shows that being a project member of a government-funded program leads to a significant positive correlation with grant success. Therefore, as an in-depth examination of the effect of human resources, we tested whether project experience in a prestigious government-funded program, often led by top researchers, would increase the likelihood of GIA success for the participants using the Japan Science and Technology Agency Strategic Basic Research Program (SBRP) as a case study [17].

**Table 3:** Relationship with the researcher with whom the participant researcher first became a project member^a^.

		Same institution, n (%)	Different institution, n (%)	Total, n (%)
**Upper level (n=47)**		38 (35.1)	9 (8.3)	47 (43.5)
	Professor	32 (29.6)	8 (7.4)	40 (37.0)
	Other than professor	6 (5.6)	1 (0.9)	7 (6.4)
Peer (n=7)		5 (4.6)	2 (1.9)	7 (6.4)
Subordinate (n=6)		3 (2.8)	3 (2.8)	6 (5.6)
Unidentified (n=1)		0 (0)	1 (0.9)	1 (0.9)
Total (N=108)		84 (77.8)	24 (22.2)	108 (100)

^a^All the projects won in the year in which each participant obtained projects for the first time were counted.

**Figure 2:**
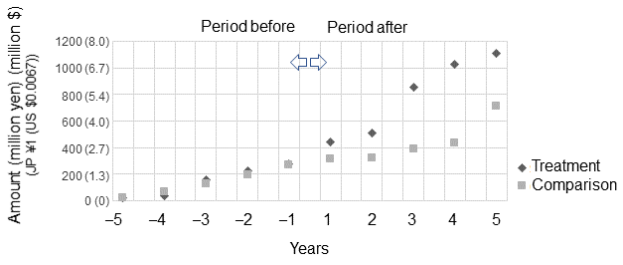
Cumulative average Grants-in-Aid award values obtained as principal investigators (PIs) before and after participating in projects in the Strategic Basic Research Program (SBRP) as project members.

Figure 2 plots the cumulative average GIA award values obtained as principal investigators over time; the 2 groups are established based on the presence or absence of SBRP experience among the 52 participants. Researchers who had been project members of the Core Research for Evolutional Science and Technology subprogram were selected as the treatment group from a total sample of 52, whereas those who had been PIs of this program were excluded. Researchers with similar *h*-index values and career lengths as those in the treatment group were then selected as the comparison group. The mean *h*-index value was 63.7 (SD 40.4) for the treatment group and 61.3 (SD 20.9) for the control group, and the mean career length was 33.4 (SD 5.2) and 25.1 (SD 5.4), respectively. The timeline was anchored on 0 for the start year of the projects in which the treatment researchers participated. For the control group, we fixed the 13th year since obtaining a doctoral degree to 0, which is the average career length at the time participants in the treatment group joined the SBRP projects (n=7 for each group).

The 2 groups begin at the leftmost point with low cumulative grant amounts. In the period leading up to the zero point, we observe no evident difference between the 2 groups, with similar funding gains. However, after participating in the SBRP, the gains of the treatment groups increased dramatically (Figure 2). By the end of the observation period, the treatment group received a higher award amount than the control group.

### Analysis 2

**Figure 3:**
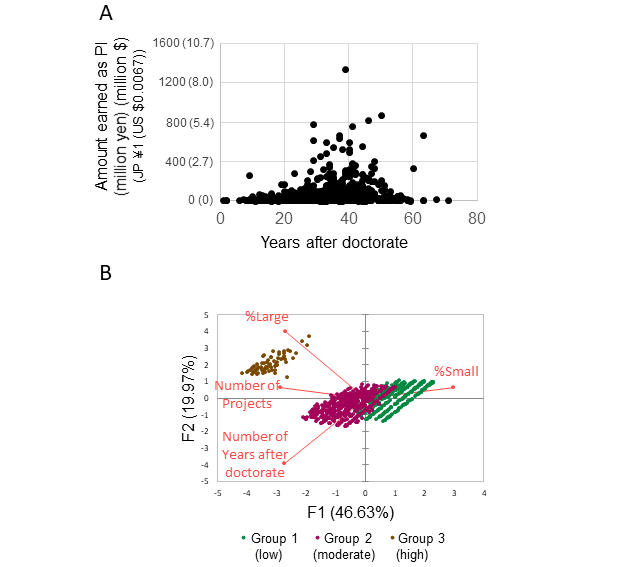
(A) Regression of the amount allocated to each participant as principal investigator (PI) against years after doctorate; includes Grants-in-Aid for Scientific Research, Grants-in-Aid for Specially Promoted Research, and Grants-in-Aid for Young Scientists. (B) Principal component analysis for the researchers’ grant success performance. Researchers were divided into 3 groups according to their performance (N=982). The term %Large indicates % large grants and the term %Small indicates % small grants.

To conduct a broader analysis of interpersonal relationships, we included 982 researchers, including 55 (5.6%) female researchers, identified as having collaborated with the researchers in the previous section (Table 1). The relationship between the number of years since obtaining a PhD and the amount earned as a principal investigator peaked at approximately 40 years after receiving the degree (Figure 3A). Using principal component analysis to evaluate each researcher’s grant success in terms of research impact, namely, the number of projects obtained, project acquisition rate for large programs, and project acquisition rate for small programs, we divided the researchers into 3 groups (Figure 3B). The term “%Small” indicates the percentage of projects from the Grant-in-Aid for Scientific Research (C) (either number of projects or amount awarded), which represents the smallest category, in the total number of projects obtained as PI. Similarly, the term “%large” indicates the Grant-in-Aid for Scientific Research (S) and Grant-in-Aid for Specially Promoted Research, which are considered large categories.

We conducted univariate logistic regression analyses to identify the factors that produced researchers with high grant success records in both large- and small-program categories (Table 4). Career length had a positive effect for large programs (odds ratio [OR] 1.07, 95% CI 1.04-1.10 for stratum 1) and a negative effect for small programs (OR 0.95, 95% CI 0.94-0.97 for stratum 1). Conversely, the variable “doctorate obtained by thesis,” which was introduced to observe the effect of age, had no significant effect. However, among the 30.8% (184/597) of researchers who received their PhD later in life, there were prominent researchers who had served as university presidents or on government committees. Earning a degree earlier or later in life did not uniformly affect a researcher’s competitiveness, and individual differences are likely to have a greater impact. Regarding differences based on sex, male researchers exhibited a negative effect on project acquisition of small programs (OR 0.29, 95% CI 0.12-0.70 for stratum 4). This indicates that male researchers are likely to move from small to larger programs.

Upon examining the impact of interaction with upper-level and peer researchers on grant success as principal investigators, we observed the following results (Table 4). More interactions with upper-level researchers resulted in fewer acquisitions of large programs (OR 0.67, 95% CI 0.50-0.89 for stratum 1) and more acquisitions of small programs (OR 1.21, 95% CI 1.07-1.36 for stratum 1) compared with the reference stratum. The differences in the ORs among the strata with different numbers of projects awarded indicated that the stronger the relationship with upper-level researchers, the higher the success rate in the smaller programs (OR 1.43, 95% CI 1.27-1.60 for stratum 4; Table 4).

After examining the impact of interaction with peer researchers separately at each stage of their careers, such as professor, associate professor, and assistant professor, we found that, in large programs, professor-professor interaction had a significant impact on the success rate of ≥2 research grants (OR 1.16, 95% CI 1.06-1.26 for stratum 2), which is not the case for the success of only 1 project (Table 4). More professor-professor interactions led to fewer acquisitions of ≥2 projects in the small category (OR 0.85, 95% CI 0.77-0.93 for stratum 2; Table 4). Interaction with peer researchers at the associate professor level and below had no significant effect on either large or small programs.

**Table 4:** Factors that affect grant success in terms of the relationship with the coresearchers (2-tailed χ^2^ test)^a^.

Variable and stratum^b^				Odds ratio (95% CI)		Significance of the model, *P* value^c^
**Number of large programs**						
	**Number of years since doctoral degree (n=982)**				<.001^d^	
		1 (n=42)	1.07^d^ (1.04-1.10)			
		2 (n=28)	1.07^d^ (1.03-1.11)			
	**Doctorate obtained by thesis (n=597)^e^**				.88	
		1 (n=16)	1.35 (0.43-4.23)			
		2 (n=13)	1.01 (0.31-3.32)			
	**Female (no; n=982)**				.82	
		1 (n=42)	0.77 (0.23-2.58)			
		2 (n=28)	1.60 (0.21-12.01)			
	**Number of upper-level researchers who designated the participants as their project members (n=982)^f^**				.003^g^	
		1 (n=42)	0.67^d^ (0.50-0.89)			
		2 (n=28)	0.72^h^ (0.53-0.99)			
	**Number of peer researchers who designated the participants as their project members (professor; n=982)^f^**				.002^g^	
		1 (n=42)	1.08 (0.98-1.19)			
		2 (n=28)	1.16^d^ (1.06-1.26)			
	**Number of peer researchers who designated the participants as their project members (associate professor; n=982)^f^**				.83	
		1 (n=42)	—^i^			
		2 (n=28)	0.69 (0.22-2.22)			
	**Number of peer researchers who designated the participants as their project members (assistant professor; n=982)^f^**				.80	
		1 (n=42)	—			
		2 (n=28)	0.56 (0.10-3.12)			
**Number of small programs**						
	**Number of years since doctoral degree (n=982)**				<.001^d^	
		1 (n=219)	0.95^d^ (0.94-0.97)			
		2 (n=190)	0.95^d^ (0.93-0.97)			
		3 (n=140)	0.96^d^ (0.94-0.98)			
		4 (n=174)	0.96^d^ (0.94-0.98)			
	**Doctorate obtained by thesis (n=597)**				.18	
		1 (n=133)	1.27 (0.77-2.10)			
		2 (n=118)	1.37 (0.81-2.31)			
		3 (n=76)	1.64 (0.88-3.06)			
		4 (n=113)	0.83 (0.50-1.37)			
	**Female (no; n=982)**				.05^h^	
		1 (n=219)	0.74 (0.28-1.96)			
		2 (n=190)	0.47 (0.19-1.18)			
		3 (n=140)	0.46 (0.17-1.23)			
		4 (n=174)	0.29^d^ (0.12-0.70)			
	**Number of upper-level researchers who designated the participants as their project members (n=982)**				<.001^d^	
		1 (n=219)	1.21^g^ (1.07-1.36)			
		2 (n=190)	1.30^d^ (1.16-1.47)			
		3 (n=140)	1.42^d^ (1.26-1.60)			
		4 (n=174)	1.43^d^ (1.27-1.60)			
	**Number of peer researchers who designated the participants as their project members (professor; n=982)**				.002^g^	
		1 (n=219)	0.94 (0.88-1.01)			
		2 (n=190)	0.85^d^ (0.77-0.93)			
		3 (n=140)	0.85^g^ (0.76-0.95)			
		4 (n=174)	0.91^h^ (0.84-0.99)			
	**Number of peer researchers who designated the participants as their project members (associate professor; n=982)**				.32	
		1 (n=219)	1.75 (0.46-2.00)			
		2 (n=190)	1.01 (0.67-1.51)			
		3 (n=140)	1.25 (0.85-1.84)			
		4 (n=174)	0.93 (0.60-1.44)			
	**Number of peer researchers who designated the participants as their project members (assistant professor; n=982)**				.57	
		1 (n=219)	1.33 (0.78-2.28)			
		2 (n=190)	1.06 (0.57-1.97)			
		3 (n=140)	1.22 (0.65-2.27)			
		4 (n=174)	1.51 (0.88-2.57)			

^a^The number of projects obtained by the participants as principal investigators was used as the dependent variable. The term *large* indicates projects from the Grant-in-Aid for Scientific Research (S) and Grant-in-Aid for Specially Promoted Research, which are considered large categories. Similarly, the term *small* indicates the Grant-in-Aid for Scientific Research category (C), which is considered the smallest category.

^b^Number of projects obtained. Cases with no corresponding acquisitions were designated as stratum 0 and used as a reference. Cases with ≥2 corresponding acquisitions were defined as stratum 2 for large categories, and cases with ≥4 acquisitions were defined as stratum 4 for the small category.

^c^Wald test (*P*<.05).

^d^*P*<.001.

^e^Doctoral degree conferred by submitting a doctoral thesis and passing its examination. We used this as a control variable and conducted an analysis by assigning a value of 0 for a regular degree and 1 for a doctorate obtained by thesis.

^f^Interaction among researchers in the same position was expressed as the number of researchers who made the participant a project member.

^g^*P*<.01.

^h^*P*<.05.

^i^No results were output by XLSTAT software for this stratum.

**Figure 4:**
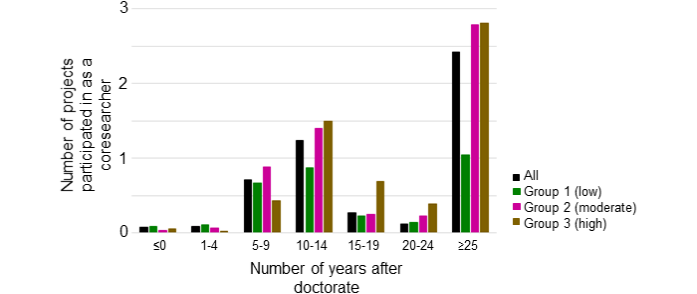
The average number of projects in which the researcher participated as a coinvestigator by number of years after obtaining a PhD and by research performance.

Figure 4 shows the number of projects in which the researcher participated as a coinvestigator based on the years after obtaining a PhD. The researchers were divided into 3 groups based on their performance level, as shown in Figure 3B. The groups with higher research achievements had a greater number of projects during the 10- to 14-year postdegree period. Group 3, the group with the highest research achievements, had the largest number of projects during the 15- to 19-year postdegree period (Figure 4). Table 5 focuses on these years as key periods and presents the average scores of several indicators of grant success and interpersonal relationships. Although the most active researchers (group 3) obtained large-program grants and interacted with more researchers during the indicated periods, the less active researchers (group 1), who primarily won small-program grants, interacted with fewer researchers during this period, many of whom were upper-level researchers (Table 5).

**Table 5:** Grant success and interaction with colleagues (2-tailed).

Group (performance)	All (n=982), mean (SE)	Group 1 (low; n=436), mean (SD)	Group 2 (moderate; n=476), mean (SD)	Group 3 (high; n=70), mean (SD)
Number of years since doctoral degree	31.19 (10.92)	26.32 (10.24)^a^	34.57 (9.95)^b^	38.51 (8.23)^c^
Number of projects obtained as a PI^d^	4.11 (2.55)	2.86 (1.71)^a^	4.79 (2.53)^b^	7.31 (2.59)^c^
Amount awarded as a PI (million yen, JP ¥^e^)	59.73 (109.49)	11.10 (7.71)	59.31 (44.57)	365.45 (215.95)
Percentage of small grants in total number of acquisitions (projects)	0.55 (0.43)	0.96 (0.14)^a^	0.25 (0.28)^b^	0.06 (0.10)^c^
Percentage of large grants in total number of acquisitions (projects)	0.02 (0.08)	0 (0)^a^	0 (0)^a^	0.24 (0.17)^b^
Number of projects participated in 10-14 y after doctorate	1.16 (1.90)	0.85 (1.38)^a^	1.40 (2.17)^b^	1.46 (2.04)^b^
Number of upper-level researchers who designated the participants as their project members (10-14 y)	0.38 (0.81)	0.40 (0.78)^a^	0.39 (0.86)^a,b^	0.19 (0.60)^b^
Number of projects participated in 15-19 y after doctorate	0.26 (1.29)	0.20 (0.84)^a^	0.24 (0.83)^a^	0.67 (3.67)^b^
Number of upper-level researchers who designated the participants as their project members (15-19 y)	0.19 (0.56)	0.27 (0.64)^a^	0.14 (0.52)^b^	0.03 (0.17)^b^

^a^*P*<.05 against groups with b and c.

^b^*P*<.05 against groups with a and c.

^c^*P*<.05 against groups with a and b.

^d^PI: principal investigator.

^e^A currency exchange rate of JP ¥1=US $0.0067 is applicable.

Table 6 presents the results of the univariate logistic regression analyses on the impact of the frequency and quality of connections using the number of project acquisitions in large and small programs as dependent variables. When the frequency of interactions during the periods was categorized by the job relationships between the partner and the participant, the results showed that interactions with peer researchers and subordinates during the 10- to 14-year postdegree period had positive effects on ≥2 large-program acquisitions (OR 1.51, 95% CI 1.09-2.09 and OR 1.31, 95% CI 1.10-1.57, respectively; Table 6). Interactions with subordinates during the 15- to 19-year postdegree period also had positive effects (OR 1.25, 95% CI 1.25-1.07). In contrast, for the small programs, interaction with upper-level researchers was important both in the 10- to 14-year and the 15 to 19-year postdegree periods, with significant positive effects (Table 6). Notably, these results show that the frequency and quality of human interaction had opposite effects on acquisitions of large and small programs.

**Table 6:** Factors that affect grant success based on the timing of experience as a project member (2-tailed χ^2^ test)^a^.

Variable and stratum^b^					Odds ratio (95% CI)		Significance of the model, *P* value^c^
**Number of large programs**							
	**Number of projects participated in 10-14 y after doctorate**						
		**Number of projects by upper-level researchers (n=968)**					.11
			1 (n=41)	0.62 (0.34-1.13)			
			2 (n=28)	0.58 (0.28-1.24)			
		**Number of projects by peer researchers (n=968)**					.04^d^
			1 (n=41)	1.12 (0.75-1.66)			
			2 (n=28)	1.51^d^ (1.09-2.09)			
		**Number of projects by subordinates (n=968)**					.007^e^
			1 (n=41)	1.15 (0.94-1.42)			
			2 (n=28)	1.31^e^ (1.10-1.57)			
	**Number of projects participated in 15-19 y after doctorate**						
		**Number of projects by upper-level researchers (n=922)**					.23
			1 (n=42)	0.32 (0.09-1.18)			
			2 (n=28)	—^f^			
		**Number of projects by peer researchers (n=922)**					.06
			1 (n=42)	1.4 (1.00-1.96)			
			2 (n=28)	1.36 (0.9-2.05)			
		**Number of projects by subordinates (n=922)**					.02^d^
			1 (n=42)	1.12 (0.94-1.33)			
			2 (n=28)	1.25^e^ (1.06-1.47)			
**Number of small programs**							
	**Number of projects participated in 10-14 y after doctorate**						
		**Number of projects by upper-level researchers (n=968)**					<.001^g^
			1 (n=210)	1.20 (0.88-1.62)			
			2 (n=186)	1.48^e^ (1.11-1.97)			
			3 (n=140)	1.88^g^ (1.42-2.49)			
			4 (n=174)	1.87^g^ (1.42-2.45)			
		**Number of projects by peer researchers (n=968)**					.12
			1 (n=210)	0.87 (0.69-1.10)			
			2 (n=186)	0.69^e^ (0.52-0.93)			
			3 (n=140)	0.81 (0.61-1.08)			
			4 (n=174)	0.80 (0.61-1.05)			
		**Number of projects by subordinates (n=968)**					.43
			1 (n=210)	1.02 (0.89-1.17)			
			2 (n=186)	0.9 (0.75-1.07)			
			3 (n=140)	0.88 (0.72-1.08)			
			4 (n=174)	0.92 (0.78-1.10)			
	**Number of projects participated in 15-19 y after doctorate**						
		**Number of projects by upper-level researchers (n=922)**					.006^e^
			1 (n=186)	1.69^d^ (1.08-2.65)			
			2 (n=178)	1.68^d^ (1.07-2.64)			
			3 (n=137)	2.06^g^ (1.32-3.22)			
			4 (n=173)	2.20^g^ (1.44-3.36)			
		**Number of projects by peer researchers (n=922)**					.04^d^
			1 (n=186)	0.90 (0.71-1.15)			
			2 (n=178)	0.60^e^ (0.44-0.83)			
			3 (n=137)	0.85 (0.64-1.12)			
			4 (n=173)	0.83 (0.64-1.09)			
		**Number of projects by subordinates (n=922)**					.17
			1 (n=186)	0.93 (0.82-1.05)			
			2 (n=178)	0.83^e^ (0.71-0.96)			
			3 (n=137)	0.94 (0.83-1.08)			
			4 (n=173)	0.93 (0.82-1.05)			

^a^The number of projects obtained by the participants as principal investigators was used as the dependent variable. The term *large* indicates projects from the Grant-in-Aid for Scientific Research (S) and Grant-in-Aid for Specially Promoted Research, which are considered large categories. Similarly, the term *small* indicates the Grant-in-Aid for Scientific Research (C), which is considered the smallest category.

^b^Number of projects obtained. Cases with no corresponding acquisitions were designated as stratum 0 and used as a reference. Cases with ≥2 corresponding acquisitions were defined as stratum 2 for the large categories, and cases with ≥4 acquisitions were defined as stratum 4 for the small category.

^c^Wald test (*P*<.05).

^d^*P*<.05.

^e^*P*<.01.

^f^No results were output by XLSTAT software for this stratum.

^g^*P*<.001.

## Discussion

### Principal Findings

This study aimed to identify factors that influence researchers’ potential to obtain external research funding by surveying their stage of career development and the types of people they interacted with using the GIA project implementation structure. Early-career interpersonal relationships, as measured using the *h*-index value of the researcher who provided the participants with their initial experience as project members, had a positive effect on grant success (Table 2). The results revealed the importance of having a good guide. We propose that a good guide can broaden project members’ perspectives by demonstrating the “behind-the-scenes” elements of effective project implementation. A good guide, not necessarily an immediate supervisor, also serves as a channel for informing fellow researchers of the perspectives required to obtain larger funds.

The results based on nonuniversity experiences (Table 2) suggest that creating an attractive proposal based solely on individual research curiosity may be difficult. The breadth of scientific expertise expressed within a research group rarely matches that expressed by an academic committee [24]. Enhancing one’s perspective by participating in large, purpose-driven projects such as those conducted by companies is important. The fact that experiencing the SBRP (a prestigious government program) as a project member facilitated subsequent grant success also confirms this hypothesis (Figure 2). A unique feature of the SBRP is that a star researcher, as the research director, conducts various interventions to modify the proposed research plan [17]. This provides the project members with opportunities to learn not only about the research conception of the principal investigator but also about the overall view of the research field held by the star researcher above the principal investigators and the strategic goals determined by policy objectives.

The effect of human connections varies depending on the career stage at which the research collaboration occurs. We found that the signs of the coefficients for the number of coresearchers were inconsistent between Multimedia Appendix 1 and Table 2. This may be due to the fact that Table 2 shows the actual relevance of the indicator to researchers’ competitiveness at the individual level, whereas Table 2 shows the macro trends. Although a larger number of collaborators indicates a larger number of projects and more grant amounts obtained in general (positive coefficient in Multimedia Appendix 1), it also suggests the importance of implementing a small number of elite projects with selected collaborators to obtain large, trend-setting projects (negative coefficient in Table 2).

Our results suggest that greater collaboration among professors increases the number of large projects obtained (Table 4). After establishing one’s specialty and becoming a professor, collaborating with researchers in different fields and leveraging synergies to obtain greater funding is easier than when one is young [25]. It is assumed that highly competitive researchers who become professors early in their careers have more opportunities to conduct collaborative research among professors, and this collaboration and friendly competition with peers may stimulate their motivation to generate new ideas worthy of being supported by large programs. Meanwhile, factors that influence midcareer grant success remain largely unexplored despite challenging expectations regarding human resource development at universities and research institutions. This study did not present significant results regarding the midcareer level (Table 4); this is because collaboration during the earlier period may include protected time until each researcher refines their research and reconciles it with that of other researchers, after which truly meaningful collaboration occurs [26,27]. Tracking the process of research development, we found that interaction with others during the periods of 10 to 14 years and 15 to 19 years after obtaining a PhD determines the size of the project that the participant will obtain (Tables 5 and 6).

This study initially attempted to identify the factors that produce researchers with high grant success records, but as grant success depends on various factors, including the assignment of reviewers and other random factors, and given cases in which initial success may have been leveraged in subsequent years [28], it was difficult to obtain clear results when focusing on large programs alone (Tables 5 and 6). Interpreted in conjunction with the results from the project acquisition rate of small programs (Tables 5 and 6), midcareer relationships that remain narrowly focused, such as immediate supervisors, keep participants’ grant success limited to small programs throughout their careers and do not lead to the acquisition of large programs (Table 6). Liu [29] pointed out that the relationship between scholar productivity and tie strength exhibits an inverted U shape using data from tourism scholars. Researchers who devote their efforts to others’ research cannot concentrate on deepening their own studies. Considering the trade-off in collaboration between acquiring ideas and paying for effort instead of undertaking part of the supervisors’ initiatives, getting involved in diverse projects by peers and subordinates is important. In particular, participating in projects by subordinates is an effective way to be exposed to the fresh ideas of a younger person and look over their projects critically as an experienced person. This will ultimately help researchers become established figures who can conduct large-scale research projects as principal investigators.

### Limitations

A limitation of this study is that we experienced some difficulties in obtaining clear data and subsequent results on the factors that influence the most prominent figures, such as those who had ≥3 projects in large programs (8/982, 0.8% in analysis 2), because of their rarity and the particular nature of their careers and research histories. These researchers tended to obtain large projects early in their careers instead of obtaining projects gradually increasing in size, which is related to the fact that they returned after international education pursuits or worked at nonuniversity institutions. Although initial success is likely to be influenced by almost uniform factors that apply to all researchers, such as publication performance relative to age, continued success is likely to be heavily influenced by individual enthusiasm and willingness to acquire large projects. Therefore, contextual analysis, such as interview surveys, will be necessary to identify the factors that produce prominent figures with outstanding achievements.

Another constraint is that the results of this analysis are limited to positive grant awards as, unlike positive awards, information on rejected projects is not publicly available. Under the Japanese grant system, the range of acceptance rates among researchers is not considered to be very large as proposals are submitted only once a year and the number of projects that can be applied for in a given year is limited by the grant system’s restrictions on duplicate applications. However, the differences among researchers with different application rates should be explored in a future study to better clarify the factors affecting researchers’ competitiveness.

### Conclusions

This study explored objective measures of success in obtaining GIA with a focus on interpersonal relationships. Our results have several implications for future research. To improve one’s ability to obtain external funding, the following are necessary aspects: developing links with channels enabling access to quality information, gaining experience in collaborative research at the midcareer stage, and developing a research area in which one can take more initiatives in the future. The function of systematically training researchers has not been sufficiently developed in the Japanese medical community, and whether one can grow as a researcher is left entirely up to the individual. Researchers must broaden the scope of their research and increase their visibility in the academic field to actualize innovative ideas. In summary, individuals should understand the power of a collaborative network and strategically choose cooperative partners.

## Appendix

Multimedia Appendix 1 Descriptive statistics and correlation matrix.

## Abbreviations

GIA: Grants-in-Aid for Scientific Research
OR: odds ratio
SBRP: Strategic Basic Research Program

## Data Availability

The data sets generated during and analyzed during this study are available from the corresponding author on reasonable request. Requests to access the data set should be directed to AH.
