# Correction: The Influence of Human Connections and Collaboration on Research Grant Success at Various Career Stages: Regression Analysis

**DOI:** 10.2196/58733

**Published:** 2024-04-05

**Authors:** Akiko Hashiguchi, Makoto Asashima, Satoru Takahashi

**Affiliations:** 1 Institute of Medicine University of Tsukuba Tsukuba Japan; 2 Advanced Comprehesive Research Organization (ACRO) Teikyo University Itabashi Japan

In “The Influence of Human Connections and Collaboration on Research Grant Success at Various Career Stages: Regression Analysis” (JMIR Form Res 2024;8:e49905) the authors noted one error.

In the “Results” section of the Abstract, the following sentence:

Tracking the process of research development, we found that collaboration during the periods of 10 to 14 years and 15 to 19 years after completing a doctorate degree determined the size of the project that the participant would obtain—interactions with peer researchers and subordinates during the 10- to 14-year postdegree period had positive effects on ≥2 large-program acquisitions (OR 1.51, 95% CI 1.09-2.09 and OR 1.31, 95% CI 1.10-1.57, respectively), whereas interactions with subordinates during the 15- to 19-year postdegree period also had positive effects (OR 1.25, 95% CI 1.25-1.07).

Has been changed to read as follows:

Tracking the process of research development, we found that collaboration during the periods of 10 to 14 years and 15 to 19 years after completing a doctorate degree determined the size of the project that the participant would obtain—interactions with peer researchers and subordinates during the 10- to 14-year postdegree period had positive effects on ≥2 large-program acquisitions (OR 1.51, 95% CI 1.09-2.09 and OR 1.31, 95% CI 1.10-1.57, respectively), whereas interactions with subordinates during the 15- to 19-year postdegree period also had positive effects (OR 1.25, 95% CI 1.06-1.47).

The correction will appear in the online version of the paper on the JMIR Publications website on April 5, 2024, together with the publication of this correction notice. Because this was made after submission to PubMed, PubMed Central, and other full-text repositories, the corrected article has also been resubmitted to those repositories.

