# Supplementary material for: The Influence of Human Connections and Collaboration on Research Grant Success at Various Career Stages: Regression Analysis
Source: JMIR Form Res. 2024 Feb 28;8:e49905. doi: 10.2196/49905 (PMC10938229; doi:10.2196/49905)
Supplement: Multimedia Appendix 1 [file formative_v8i1e49905_app1.docx]

**Supplemental Table 1.** Descriptive statistics and correlation matrix (N=52).

| Variable | | 1. The sum of the maximum allocated amount of projects obtained as principal investigator^a^ | 2. Number of years since doctoral degree | 3. Sex | 4. Nonuniversity institutions | 5. University rank (graduate)^b^ | 6. University rank (currently affiliated)^b^ | 7. Total allocation in early stage^c^ | 8. Number of coresearchers connected with through projects | 9. Betweenness centrality | 10. *h*-index of the researcher with whom the participant researcher first became a project member | 11. Number of papers | 12. Number of first-authored papers | 13. Number of last-authored papers | 14. Number of government-funded programs participated in as a project member | 15. Total *h*-indexes of researchers interacted with through other government-funded programs^d^ |
| --- | --- | --- | --- | --- | --- | --- | --- | --- | --- | --- | --- | --- | --- | --- | --- | --- |
| **1. The sum of the maximum allocated amountof projects obtained as principal investigator (mean 13,394.2, SD 15,023.6; range 1500-70,000)** | | | | | | | | | | | | | | | | |
|  | *r* | 1.000 | 0.109 | −0.247 | 0.377 | −0.192 | −0.531 | 0.272 | 0.361 | 0.355 | 0.192 | 0.521 | 0.258 | 0.628 | 0.569 | 0.606 |
|  | *P* value^e^ | —^e^ | — | — | <.05 | — | <.05 | — | <.05 | <.05 | — | <.05 | — | <.05 | <.05 | <.05 |
| **2. Number of years since doctoral degree (mean 29.6, SD 6.0; range 17-41)** | | | | | | | | | | | | | | | | |
|  | *r* | 0.109 | 1.000 | 0.027 | 0.151 | 0.151 | 0.015 | −0.453 | 0.510 | 0.392 | −0.096 | 0.044 | −0.214 | 0.263 | −0.046 | 0.028 |
|  | *P* value | — | — | — | — | — | — | <.05 | <.05 | <.05 | — | — | — | — | — | — |
| **3.** Sex **(mean 0.0, SD 0.2; range 0-1)** | | | | | | | | | | | | | | | | |
|  | *r* | −0.247 | 0.027 | 1.000 | −0.049 | 0.036 | 0.112 | −0.220 | −0.197 | −0.207 | −0.003 | −0.307 | −0.130 | −0.163 | −0.199 | −0.196 |
|  | *P* value | — | — | — | — | — | — | — | — | — | — | <.05 | — | — | — | — |
| **4. Nonuniversity institutions (mean 0.1, SD 0.2; range 0-1)** | | | | | | | | | | | | | | | | |
|  | *r* | 0.377 | 0.151 | −0.049 | 1.000 | −0.062 | −0.148 | −0.037 | 0.055 | 0.008 | 0.016 | 0.151 | 0.033 | 0.291 | 0.125 | 0.194 |
|  | *P* value | <.05 | — | — | — | — | — | — | — | — | — | — | — | <.05 | — | — |
| **5. University rank (graduate; mean 2.3, SD 1.7; range 1-7)** | | | | | | | | | | | | | | | | |
|  | *r* | −0.192 | 0.151 | 0.036 | −0.062 | 1.000 | 0.169 | 0.156 | 0.059 | −0.108 | −0.146 | −0.080 | −0.015 | −0.172 | −0.251 | −0.215 |
|  | *P* value | — | — | — | — | — | — | — | — | — | — | — | — | — | — | — |
| **6. University rank (currently affiliated; mean 4.4, SD 1.9; range 1-7)** | | | | | | | | | | | | | | | | |
|  | *r* | −0.531 | 0.015 | 0.112 | −0.148 | 0.169 | 1.000 | −0.205 | −0.238 | −0.236 | −0.384 | −0.543 | −0.344 | −0.468 | −0.488 | −0.506 |
|  | *P* value | <.05 | — | — | — | — | — | — | — | — | <.05 | <.05 | <.05 | <.05 | <.05 | <.05 |
| **7. Total allocation in early stage (mean 2341.1, SD 5438.3; range 0-35,490)** | | | | | | | | | | | | | | | | |
|  | *r* | 0.272 | −0.453 | −0.220 | −0.037 | 0.156 | −0.205 | 1.000 | 0.013 | 0.030 | 0.236 | 0.216 | 0.322 | 0.153 | 0.054 | 0.132 |
|  | *P* value | — | <.05 | — | — | — | — | — | — | — | — | — | <.05 | — | — | — |
| **8. Number of coresearchers connected with through projects (mean 54.9, SD 54.5; range 0-278)** | | | | | | | | | | | | | | | | |
|  | *r* | 0.361 | 0.510 | −0.197 | 0.055 | 0.059 | −0.238 | 0.013 | 1.000 | 0.863 | 0.123 | 0.531 | 0.159 | 0.546 | 0.312 | 0.458 |
|  | *P* value | <.05 | <.05 | — | — | — | — | — | — | <.05 | — | <.05 | — | <.05 | <.05 | <.05 |
| **9. Betweenness centrality (mean 0.0, SD 0.0; range 0-0)** | | | | | | | | | | | | | | | | |
|  | *r* | 0.355 | 0.392 | −0.207 | 0.008 | −0.108 | −0.236 | 0.030 | 0.863 | 1.000 | 0.094 | 0.559 | 0.196 | 0.476 | 0.418 | 0.541 |
|  | *P* value | <.05 | <.05 | — | — | — | — | — | <.05 | — | — | <.05 | — | <.05 | <.05 | <.05 |
| **10. *h*-index of the researcher with whom the participant researcher first became a project member (mean 56.9, SD 42.6; range 0-247)** | | | | | | | | | | | | | | | | |
|  | *r* | 0.192 | −0.096 | −0.003 | 0.016 | −0.146 | −0.384 | 0.236 | 0.123 | 0.094 | 1.000 | 0.254 | 0.193 | 0.173 | 0.134 | 0.297 |
|  | *P* value | — | — | — | — | — | <.05 | — | — | — | — | — | — | — | — | <.05 |
| **11. Number of papers (mean 144.9, SD 138.1; range 19-914)** | | | | | | | | | | | | | | | | |
|  | *r* | 0.521 | 0.044 | −0.307 | 0.151 | −0.080 | −0.543 | 0.216 | 0.531 | 0.559 | 0.254 | 1.000 | 0.551 | 0.749 | 0.740 | 0.677 |
|  | *P* value | <.05 | — | <.05 | — | — | <.05 | — | <.05 | <.05 | — | — | <.05 | <.05 | <.05 | <.05 |
| **12. Number of first-authored papers (mean 13.6, SD 11.0; range 2-56)** | | | | | | | | | | | | | | | | |
|  | *r* | 0.258 | −0.214 | −0.130 | 0.033 | −0.015 | −0.344 | 0.322 | 0.159 | 0.196 | 0.193 | 0.551 | 1.000 | 0.413 | 0.426 | 0.368 |
|  | *P* value | — | — | — | — | — | <.05 | <.05 | — | — | — | <.05 | — | <.05 | <.05 | <.05 |
| **13. Number of last-authored papers (mean 44.2, SD 49.5; range 0-330)** | | | | | | | | | | | | | | | | |
|  | *r* | 0.628 | 0.263 | −0.163 | 0.291 | −0.172 | −0.468 | 0.153 | 0.546 | 0.476 | 0.173 | 0.749 | 0.413 | 1.000 | 0.561 | 0.545 |
|  | *P* value | <.05 | — | — | <.05 | — | <.05 | — | <.05 | <.05 | — | <.05 | <.05 | — | <.05 | <.05 |
| **14. Number of government-funded programs participated in as project member (mean 4.1, SD 4.9; range 0-21)** | | | | | | | | | | | | | | | | |
|  | *r* | 0.569 | −0.046 | −0.199 | 0.125 | −0.251 | −0.488 | 0.054 | 0.312 | 0.418 | 0.134 | 0.740 | 0.426 | 0.561 | 1.000 | 0.787 |
|  | *P* value | <.05 | — | — | — | — | <.05 | — | <.05 | <.05 | — | <.05 | <.05 | <.05 | — | <.05 |
| **15. Total *h*-indexes of researchers interacted with through other government-funded programs (mean 81.6, SD 123.9; range 0-532)** | | | | | | | | | | | | | | | | |
|  | *r* | 0.606 | 0.028 | −0.196 | 0.194 | −0.215 | −0.506 | 0.132 | 0.458 | 0.541 | 0.297 | 0.677 | 0.368 | 0.545 | 0.787 | 1.000 |
|  | *P* value | <.05 | — | — | — | — | <.05 | — | <.05 | <.05 | <.05 | <.05 | <.05 | <.05 | <.05 | — |

^a^The analysis includes *Grants-in-Aid for Scientific Research (S)*, *(A)*, *(B)*, or *(C)*; *Grants-in-Aid for Specially Promoted Research*; and *Grants-in-Aid for Young Scientist (S)* or *(A)*.

^b^The variables were quantified based on The Times Higher Education World University Rankings 2023 and have 7 levels of classification, with a higher ranking having a smaller numerical value, that is, 1 for the top 100 ranking, 2 for 201 to 600, a value of 3 for 601 to 1000, a value of 4 for 1001 to 1200, a value of 5 for 1201 to 1500, a value of 6 for ≤1501, and 7 for out of ranking.

^c^Total allocation earned as a principal investigator during the first 10 years after obtaining a PhD.

^d^The sum of *h*-indexes of researchers who included the participant researcher as a project member.

^e^ *P*-values greater than 0.05 are represented by em dashes.
